# New Finds and Ecology of the Rare Liverworts *Scapania apiculata*, *Scapania carinthiaca,* and *Scapania scapanioides* in Austria

**DOI:** 10.3390/plants12152753

**Published:** 2023-07-25

**Authors:** Michaela Kropik, Harald G. Zechmeister

**Affiliations:** Department of Botany and Biodiversity Research, University of Vienna, Rennweg 14, 1030 Vienna, Austria; michaela.kropik@univie.ac.at

**Keywords:** bryophytes, epixylic, deadwood, climate, Habitats Directive, IUCN Red List of Threatened Species, conservation

## Abstract

*Scapania apiculata*, *Scapania carinthiaca*, and *Scapania scapanioides* are rare deadwood-dwelling liverworts threatened across Europe. *Scapania carinthiaca* is thus listed in the Habitats Directive. However, their distribution data are scattered, and their ecologic demands are insufficiently studied. Here, we present new locations and data on the ecology of the species, which resulted from a targeted search in selected regions of Austria. We found ten new sites each for *Scapania apiculata* and *Scapania scapanioides* and twenty for *Scapania carinthiaca*. Reproduction was exclusively asexual. The macroclimates of all known locations in Austria did not differ significantly between the three species. It was consistently wet, with a mean annual precipitation of 1615.3 mm, a high evenness of rainfall, and a low desiccation risk. The mean temperature averaged 7.4 °C. The habitat was shaded dead wood of *Picea abies*, *Abies alba*, and *Fagus sylvatica* of all decay stages at a median distance of 2.5 m from streams or springy areas in semi-natural forests of montane and submontane regions. Thus, high deadwood volumes under a suitable climate are a prerequisite for the occurrences of the species. The number of locations of new finds has more than doubled in Austria and thus in Europe.

## 1. Introduction

*Scapania apiculata* Spruce, *Scapania carinthiaca* J.B.Jack ex Lindb., and *Scapania scapanioides* (C. massal.) Grolle are three rare deadwood-dwelling liverworts of exceptional smallness within the species-rich genus *Scapania* (Scapaniaceae, Jungermanniales), which still raises taxonomic questions [1,2]. With a size of only a few millimetres, the three species are easy to overlook, and a targeted search is like looking for a needle in a haystack.

These are north-temperate species, but their range is still insufficiently known [3]. Reports to date have come mainly from the mountains of Central Europe, Scandinavia, and North America [4,5]. In Austria, there are 16 published records of *S. carinthiaca*—many of which are in the south of Austria, in Carinthia—ten for *S. apiculata*, and five for *S. scapanioides* [6]. In the neighbouring countries Germany and Liechtenstein, *S. apiculata* is expected to occur but without endangerment classifications [7]; the Czech Republic classifies the species as critically endangered [8]; in Switzerland, it has the status endangered; Italy and Hungary state insufficient distribution data; in Slovenia, there are doubts about its occurrence [7,9]; and in Slovakia, it is classified as least concern [10]. *S. carinthiaca* is regionally extinct in Slovakia [10]; critically endangered in Germany [11], Italy, the Czech Republic, and Slovenia [7]; and endangered in Liechtenstein [12] and Switzerland [7]. *S. scapanioides* is known to occur only in Italy and Switzerland. There, it is classified as endangered [7]. The classifications for Europe [3,7] for *S. apiculata* (LC), *S. carinthiaca* (EN), and *S. scapanioides* (CR) might thus change due to an improved data situation in the future.

The reported habitat for the European sites is deadwood in humid, shady areas; the colonised logs are mostly near mountain streams, in ravines, or on deadwood in mountain forests influenced by slope pressure water [6,13,14,15]. Except for *Scapania carinthiaca*, there are also reports of the species on siliceous rocks near streams [6].

Overall, the data on this species group are incomplete and partly contradictory. Since *S. carinthiaca* is a species of Annex II of the Habitats Directive and thus subject to monitoring and reporting obligations, knowledge has improved the data situation for the entire species group to some extent, but studies on their ecology are lacking. Therefore, this study aimed to deepen the knowledge of the distribution and ecological requirements of the target species in Austria so that future surveys can be more targeted.

## 2. Results

### 2.1. Distribution and Ecology

**Figure 1:**
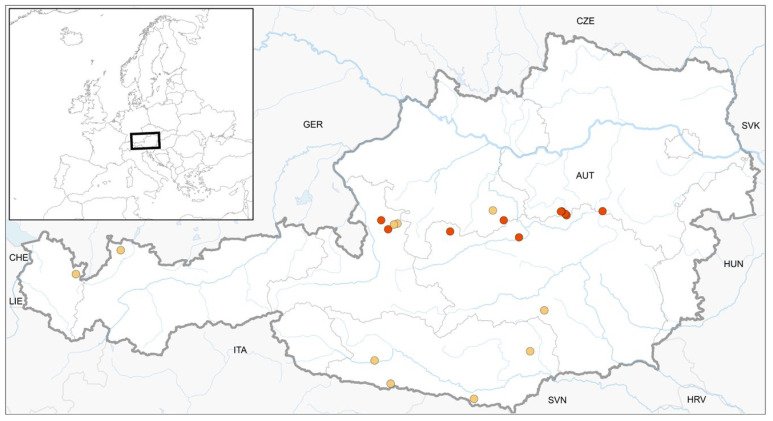
Location of ten previously published finds (yellow, see Appendix A, Table A2) and ten new finds (red) of *S. apiculata* in Austria.

**Figure 2:**
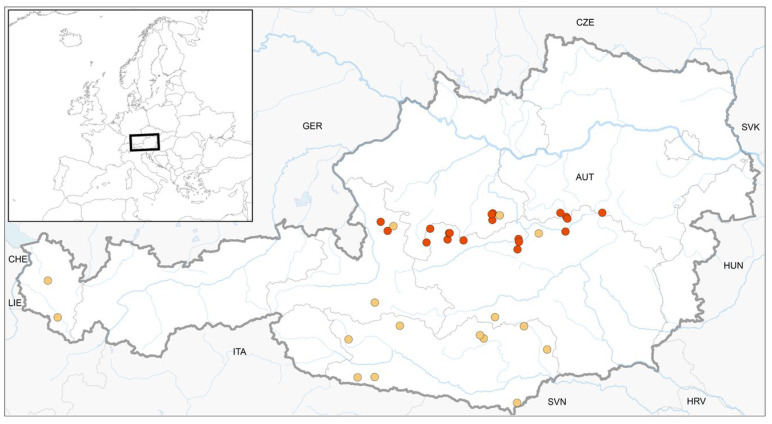
Location of 16 previously published finds (yellow) and 20 new finds (red) of *S. carinthiaca* in Austria.

**Figure 3:**
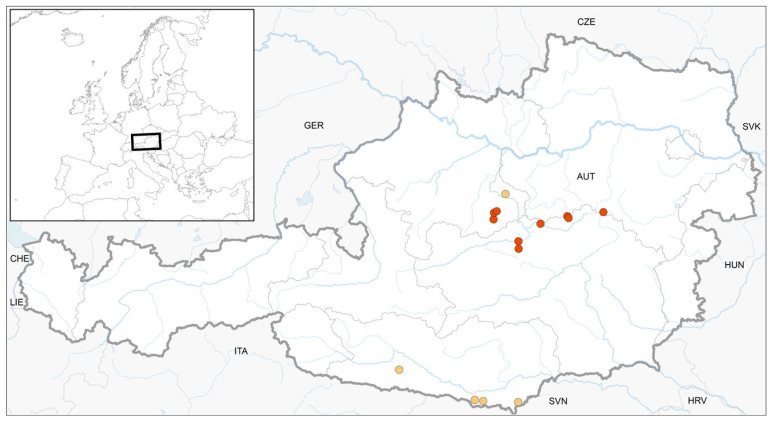
Location of 5 previously published finds (yellow) and 10 new finds (red) of *S. scapanioides* in Austria.

In total, we found 17 colonised deadwood logs at ten study sites with *S. apiculata* (Figure 1), 29 deadwood logs at twenty study sites with *S. carinthiaca* (Figure 2), and 11 deadwood logs at ten study sites with *S. scapanioides* (Figure 3). In several cases, the three species co-occurred. According to the project regions, study sites were located in the Alpine region of Styria, Salzburg, Lower and Upper Austria (see Appendix A, Table A1 for details), with a distribution focus in the Northern Limestone Alps.

**Figure 4:**
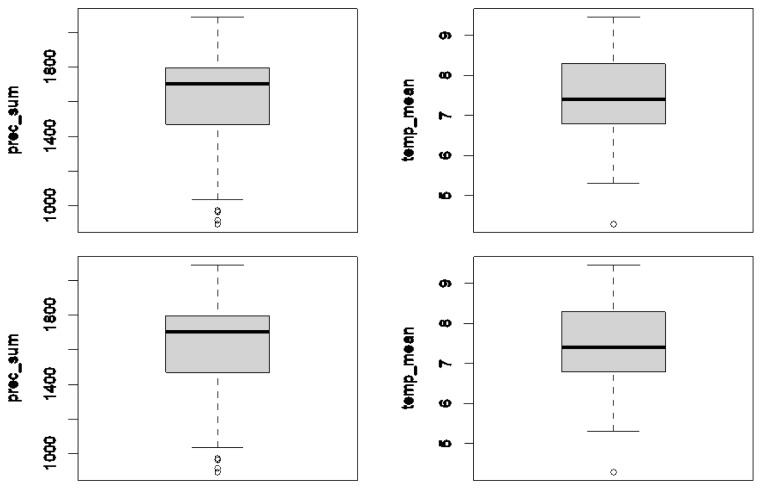
Climatic conditions at the Austrian locations of *S. apiculata*, *S. carinthiaca*, and *S. scapanioides*: boxplots of mean annual precipitation sum (prec_sum), mean annual temperature (temp_mean) in °C, evenness of precipitation (daysprec11), and desiccation risk (p0cont_gt_20) based on 71 finds (including previously published ones) across Austria.

The macroclimates in the Austrian sites known so far did not differ significantly between the three species regarding the mean annual temperature (H(2) = 0.124; *p* = 0.54), mean annual precipitation sum (H(2) = 1.49; *p* = 0.47), desiccation risk (H(2) = 2.49; *p* = 0.29), or evenness of precipitation (H(2) = 0.62; *p* = 0.73). Therefore, in Figure 4, we present the climate indicators for all new and previously published sites in one boxplot. The macroclimate was consistently wet, with a mean annual precipitation of 1615.3 mm. The mean annual temperature averaged 7.4 °C. The precipitation distribution showed a high evenness, with at least one millimetre of rain falling on an average on more than 126 days per year. At the same time, the risk of desiccation in the growing sites was low; the maximum number of precipitation-free days with a temperature of more than 20 degrees averaged 5.9 days.

Within this distinctly wet macroclimate, the microclimate was also humid and reduced the risk of desiccation. Growth sites were on lying deadwood shaded all day long directly adjacent to streams in headwaters (trout region) in ravines or north-exposed forests influenced by spring areas or slope water. The median distance to the water course was 2.5 m. Thus, the local risk of desiccation in the place of growth was low to unlikely.

**Figure 5:**
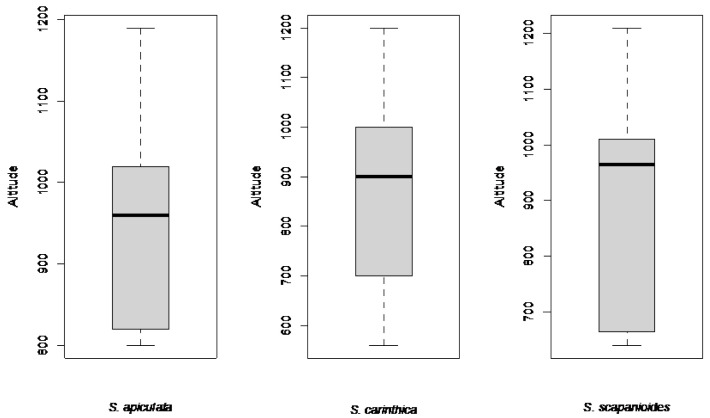
Boxplots showing the altitude of Austrian locations of *S. apiculata*, *S. carinthiaca*, and *S. scapanioides*.

Regarding altitude, the finds came from the montane to submontane region (Figure 5), with a few exceptions.

The logs of the host trees had diameters of at least 30 cm, but most were significantly larger. Host trees showed decay stages from 2 to 5 except but did not show decay stage 1 with still-intact bark. Most finds of *S. carinthiaca* were from *Picea abies*, and some finds were from *Fagus sylvatica* and *Abies alba*. *S. apiculata* occurred relatively evenly on *Picea abies*, *Abies alba*, and *Fagus sylvatica*; *S. scapanioides* mainly occurred on *Picea abies* and rarely occurred on *Abies alba*.

### 2.2. Characteristics of the Target Species

As is characteristic of the genus, the leaves of the target species consist of a smaller dorsal and a larger ventral lobe. In the target species, the lobes enclose the stem in a sheathing manner and do not run down it. The leaf bases are not keeled. Characteristically, single- or two-celled gemmae, depending on the species, serve for vegetative reproduction. They are located either on the leaf margins or erect flagellar shoots. The perianth mouth is entire, but a perianth is rarely present. Usually, large parts of the stem are covered densely with long rhizoids.

**Figure 6:**
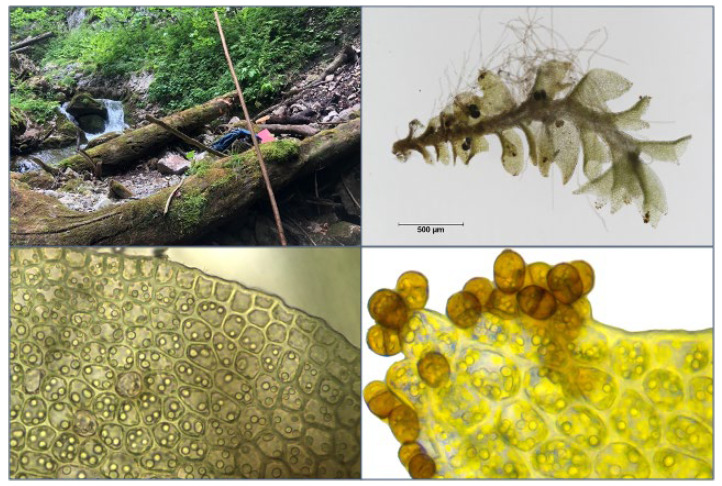
Photographs of *S. scapanioides* in situ (**top left**), a single plant (**top right**), its cells (**bottom left**), and its bicellular, brown gemmae (**bottom right**).

**Figure 7:**
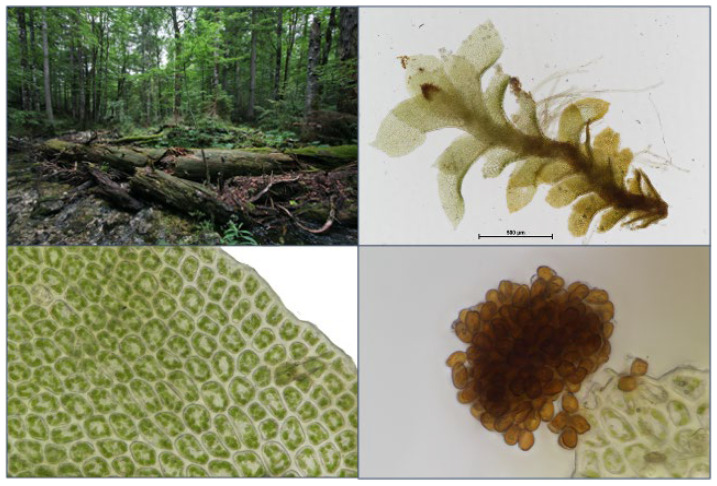
Photographs of a characteristic location of *S. carinthiaca* on deadwood (**top left**); a single plant (**top right**); its cells, with characteristically unequal size and slightly bulging leaf margin (**bottom left**); and its unicellular brown gemmae (**bottom right**).

**Figure 8:**
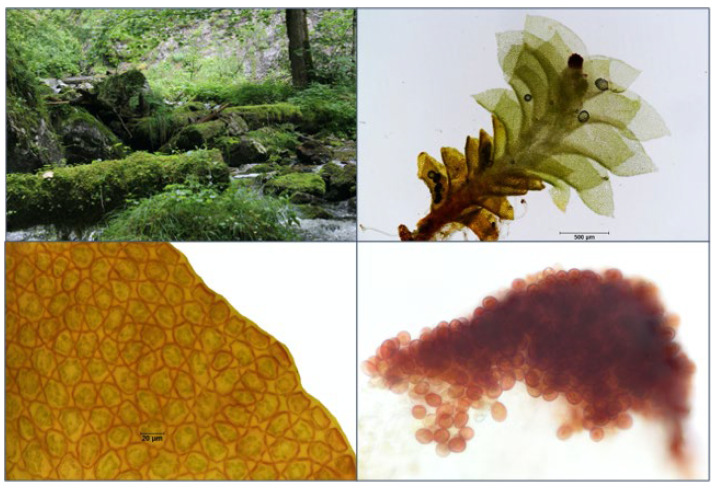
Photographs of *S. apiculata* in situ (**top left**); a single plant (**top right**); its characteristic cells, with strongly triangularly thickened cell corners (**bottom left**); and the unicellular gemmae (**bottom right**).

A first delimitation of the three target species within the genus is allowed by the location: the tiny target species, of only a few millimetres in size, occur almost exclusively on lying deadwood at wet sites, except for *S. carinthiaca*, which was found sporadically on wet silicate rock. Gemmae are advantageous for reliable identification, especially for distinguishing the target species from the juvenile and vestigial forms of other species within the genus *Scapania*. All our finds had abundant gemmae. However, we never observed a perianth. *S. scapanioides* can be distinguished from the other two species by its two-celled, broadly oval gemmae of brown colour (Figure 6). However, *S. carinthiaca* and *S. apiculata* have unicellular gemmae. They are brown in *S. carinthiaca* (Figure 7) and reddish-brown to black in *S. apiculata* (Figure 8). The gemmae of *S. apiculata* often sit at the tip of erect small-leaved shoots or, as observed in *S. carinthiaca*, at the leaf margins A clear differentiation of *S. apiculata* from *S. carinthiaca* is made possible by the triangularly thickened cell corners of *S. apiculata* (Figure 8). Furthermore, *S. carinthiaca* has conspicuously pointed upper lobes, which are pronouncedly smaller than the lower lobes at the shoot tips and are oriented towards the shoot tip (Figure 7); by contrast, the rectangular upper and lower lobes in *S. apiculata* are nearly equal in size. Table 1 summarises the particularly well-differentiated characteristics of the target species.

## 3. Discussion

### 3.1. Distribution and Ecology

The present data improve the knowledge about the distribution of the three species in Austria by providing ten new records each for *S. apiculata* and *S. scapanioides* and twenty new records for *S. carinthiaca*. The *S. carinthiaca* finds known so far have mainly been in Carinthia [6], where the species was first described [4] and to which the epithet refers. However, the Northern Limestone Alps seem to be another centre of its distribution (Figure 1, Figure 2 and Figure 3), as the current surveys showed. *S. apiculata* and *S. scapanioides* seem to have a similar distribution. However, it is still not fully clarified. Targeted searches in individual provinces, such as Tyrol, lead us to expect further finds. The small species are easy to overlook in quadrant-based mappings and occurred only in small populations, as was observed by others [5]. Therefore, we recommend targeted searches.

Concerning habitat, the three target species in Austria occur predominantly in sub-montane to montane regions [6], as confirmed by this study (Figure 5). All our finds of the target species were on deadwood, as Müller [4] also stated. However, isolated records of *S. carinthiaca* from wet silicate rocks occur in Austria [6]. In general, the substrate was acidic, as observed by others [5], with flooding increasing the base content [6]. The species have been found on peat on rock walls in North America [5] and on softwood in Scandinavia [16], but they have not been found in these habitats in Austria. Apart from *Fagus sylvatica*, the three target species we found mainly on the coniferous wood of the host trees *Picea abies* and *Abies alba* in the upper reaches of streams. Lying deadwood of early decay stages at a median distance of 2.5 m from a stream seems ideal for colonization under the prevailing conditions in Austria. Regular flooding, which might increase the base content of the deadwood logs, seems to be a prerequisite for the target species [6]; however, very wet, permanently flooded deadwood seems unsuitable as a habitat. We observed microfilms under such conditions, which could hinder the establishment of the target species, as was also observed in deadwood communities [17].

The terpenoids observed in *Scapania carinthiaca* [18] might favour survival under these wet conditions. They are also known to occur on other bryophyte species [19] and serve in other biotic and pathogenic interactions, such as interactions with fungi, which presumably benefit from the extremely humid conditions. However, no studies in this regard are available for the target species. Likewise, the fungal growths visible in cross-section, as also described by Schuster [5], have not yet been studied in detail; such studies could provide further information on moss–fungus interactions.

There are probably also physiological reasons why excessively wet conditions have an unfavourable effect on the occurrence of the target species. In water, gas diffusion is decreased by a factor of about 104 compared with that in air, and thus the respiration and photosynthesis rates of poikilohydric moss plants are also reduced [20]. Many finds have originated from logs of decay stage two, on which competition from fast-growing pleurocarpous species is still low. Vigorous species might hinder the establishment of the target species on logs of later decay stages. Beyond that, however, the species persist to late decomposition stages.

In addition to an adequate substrate, however, a macroclimate with high precipitation is a prerequisite for the occurrence of the target species. High and evenly distributed rainfall reduces the risk of desiccation, which is limiting for moist-adapted deadwood liverworts [21]. In addition, the location of the microsites on shaded, permanently moist deadwood, predominantly along streams, further reduces the risk of desiccation. The average number of warm days (over 20 °C) in a row without precipitation was 5.9 days, and desiccation was thus low. These results were consistent with the findings on *Buxbaumia viridis* (Moug. ex Lam. & DC.) Brid. ex Moug. & Nestl. [22] and other forest bryophytes, for which, depending on the species, desiccation of four to ten days is limiting [22,23].

As a corollary, favourable conditions for occurrence include high deadwood volumes with a low desiccation risk and consistently high (air) humidity along streams, as well as all-day shading of the growing site, as is the case in narrow gorges, for example. Some finds have been from mountain forests with very high precipitation, where deadwood logs influenced by slope water may provide suitable habitats. According to current data, Austria is highly responsible for all three species.

### 3.2. Implications for Management and Conservation

According to current knowledge, the rarest of the three species is *S. scapanioides*, as assumed for Europe, where the IUCN [15] estimated no more than 50 individual equivalents, although it admitted that the distribution was poorly clarified. The threat categories for the Austrian provinces (Table 2) need to be adjusted according to the latest data from this study and supplemented by the new finding of *S. scapanioides* in Lower Austria.

Most of the Austrian occurrences are in protected areas (see Annex), which is advantageous because the target species are strongly dependent on natural dynamics, which, outside protected areas, often collide with flood protection or power plants.

The three *Scapania* species are weak in competition and settle on deadwood in low to middle decomposition stages, where the competition from fast-growing mosses and vascular plants is negligible. Due to the decomposition process, succession to more vigorous species, especially mosses, can be observed [28,29], and these species often overgrow the tiny *Scapania* species, as observed by others [30].

Apart from the competition, a deadwood log has a finite lifespan and is subject to high site dynamics along streams. Flooding causes deadwood logs to be relocated or completely uprooted from favourable sites. A sufficient supply of potentially colonisable deadwood logs is, therefore, a prerequisite for the long-term survival of the target species. They must be able to compensate for local extinction on a single deadwood log by successfully colonising elsewhere. Currently known populations will thus disappear in the future, together with their habitat, while ideally, suitable growth sites will emerge elsewhere and be successfully colonised. These population dynamics require large amounts of deadwood of larger diameters; deadwood of smaller diameters was populated only in exceptional cases, as others have also reported [29,31]. An important factor is the continuous shading and thus low desiccation risk of the host tree logs, which is less of a problem in narrow ravines and under closed canopy covers in forests. The increase in beetle calamities or windthrows and the loss of old-growth forests and virgin forests throughout Europe [32] are thus threats to the three target species. Management should thus consist of maintaining or restoring semi-natural forests along mountain streams and leaving deadwood of larger diameters, especially along shady stream margins, preferably in narrow gorges. The same applies to deadwood bryophytes in general, for which 60 m^3^ of lying deadwood per hectare with a diameter over 30 cm was the critical threshold for species-rich communities [22]. This measure also fosters the biodiversity of other deadwood-related species groups [33,34,35,36]. Softwoods, such as willow observed in Norway [16], do not seem to be an adequate substrate under the conditions prevailing in Austria.

In addition, the persistence of a population depends highly on the successful formation of reproductive units. Gemmae were found at all sites, but no perianths were found. Vegetative reproduction thus appears to be the main form of reproduction in the three target species. Asexual reproduction is generally widespread in temperate liverworts, especially in harsh environments [37]. This has been considered a means of maintaining local populations [37], as gemmae have higher survival rates than spores due to their size, and they germinate faster than the latter [38]. However, asexual reproduction reduces the spatial scale of dispersal in terrestrial environments [38], but this should not adversely affect the target species, as their gemmae are most likely dispersed downstream by water. However, asexual reproduction may help the target species maintain well-adapted phenotypes in an ecologically stable habitat [39]. The factors that control the formation of reproductive units have not yet been studied in detail for the target species. Concerning metapopulation ecology, vital populations in the upper reaches could act as source populations to supply lower-lying growing sites with diaspores [40,41]. Water is most likely responsible for the dispersal of the gemmae of the target species. Compared with terrestrial habitats, where dispersal capacity is usually limited to a few centimetres [38,42,43], water overcomes long distances; however, dispersal remains confined further down the watercourse, and the contribution of a coincidence to finding an adequate deadwood log is very high. The extent to which animal vectors, as assumed in a few previous studies [44,45], are relevant for dispersal may also play a role in the target species’ colonisation of new stream systems, and concerning upstream dispersal, this requires a focused investigation. Dispersal by spores seems to occur only in exceptional cases, at least in the Austrian growing sites. Thus, dispersal limitation could be an influencing factor for the target species but requires further investigation.

As a corollary, it takes much deadwood under a suitable climate in the upper reaches of mountain streams, often removed outside protected areas due to the risk of clogging. To further improve data, targeted searches are advantageous. Monitoring should take into account natural population dynamics.

## 4. Materials and Methods

We collected data between 2012 and 2022 within several projects [46,47,48,49]. The study on deadwood bryophytes [49] represented Austria in terms of its climatic gradient and different available deadwood volumes of forest sites. It followed a standardized random method [17] and first brought baseline data on the distribution and ecological requirements of the target species. On the basis of these and knowledge from previous locations in Austria [6], targeted searches were carried out in further projects [46,47,48] focused on the occurrence of species listed in Annex II of the Habitats Directive, including *S. carinthiaca*. After selecting potential locations on the basis of our previous experiences, preliminary mapping, and terrain data, we specifically searched numerous deadwood logs for the occurrence of the target species in these areas. The target regions of the projects were the provinces of Lower Austria, Styria, and the Kalkalpen National Park in Upper Austria. Searches in previously published locations in Austria were not carried out for this study, as these locations are recent. We thus assumed that the species still occurred there.

M.K. and H.Z. performed the field surveys and microscopic identification of species. Specimens are in private herbaria. The nomenclature of the bryophytes followed the Austrian Checklist of Bryophytes [50]. Because of the comparably small number of finds, the target species still raise taxonomic questions that remain incompletely resolved, and species concepts are partly contradictory, e.g., [4,5,51,52]. Buch [53] placed Scapaniella as a separate genus of tiny *Scapania* species without distinct stem cortex differentiation, within which he distinguished *S. apiculata* as a separate section. Crandall-Stotler et al. [54] also placed Scapaniella as a genus. Müller, on the other hand, placed *S. apiculata* and *S. carinthiaca* in the group Scapaniella alongside *S. massalongii* and *S. glaucocephala* but did not consider a separate genus of Scapaniella to be justified because its characteristics correspond to the genus Scapania. Like Potemkin [55] and Köckinger [6], we synonymised *C. carinthiaca* and *C. massalongii*. Likewise, we considered *S. glaucocephala* to be synonymous with *S. scapanioides*, as others have proposed [2,6].

We extracted the climate data for historical and new sites from the SPARTACUS gridded dataset [56,57] of the Central Institute for Meteorology and Geodynamics (ZAMG). It has a spatial resolution of one square kilometre and a temporal resolution of one day between 2004 and 2018. On the basis of these data, we calculated the mean annual temperature (temp_mean) in °C and mean annual precipitation sum (prec_sum). We aimed to depict the evenness of precipitation (daysprec11) by calculating the number of days with at least one millimetre of precipitation and a mean temperature above 0 °C since bryophytes benefit more from regular (even light) rain than from isolated heavy rain events [58]. As an approximation of desiccation risk [21], we calculated the maximum number of consecutive days without precipitation whose mean daily temperature was more than 20 °C (p0cont_gt_20) since desiccation is a limiting factor for moisture-adapted forest bryophytes [23,59,60], the deadwood specialist *Buxbaumia viridis* [22], and species-rich deadwood communities in general [17]. We reported the decay stage of host tree logs in a five-part scale following the Swiss Forest Inventory [61,62]: Decay stage 1 describes sap-bearing fresh wood with the bark still intact; decay stage 2 stands for sapless, firm hardwood that is difficult to penetrate with a pocket knife in the direction of the grain. In rotten wood of decay stage 3, this is easily possible in the direction of the grain but not across it; in soft rotten wood of decay stage 4, this is possible regardless of grain direction with only a little pressure. Decay stage 5 describes mulch wood that is barely coherent and very loose. We generated statistics, including the boxplots, using R statistical software [63], version 4.0.3. If the locations of the three species differed regarding the given climate variables, we performed a Kruskal–Wallis test.

## Figures and Tables

**Table 1:** Well-differentiated characteristics of the deadwood-dwelling liverworts *S. apiculata*, *S. carinthiaca*, and *S. scapanioides*.

	* S. apiculata *	* S. scapanioides *	* S. carinthiaca *
Cell corners	Strikingly triangular thickened	Not thickened	Not thickened
Gemmae	Unicellular reddish to black	Bicellular brown	Unicellular brown

**Table 2:** Status of *S. apiculata*, *S. carinthiaca*, and *S. scapanioides* in the Red Data Lists of the Austrian provinces Carinthia [24], Lower Austria [25], Upper Austria [26], and Vorarlberg [27] and Red List Status for Austria and Europe [3] based on the IUCN categories: CR—Critically Endangered, EN—Endangered, NT—Near Threatened, VU—Vulnerable; °—Occurrence of the species confirmed (either least concern or no information about status).

	Carinthia	Lower Austria	Upper Austria	Vorarlberg	Austria	Europe
* S. apiculata *	EN	CR	EN	-	VU	NT
* S. carinthiaca *	VU	CR	EN	CR	°	EN
* S. scapanioides *	EN	-	EN	-	VU	CR

## Data Availability

Not applicable.

## Appendix A

**Table A1:** New reports of *S. apiculata*, *S. carinthiaca*, and *S. scapanioides* in Austria.

	Species	Finder	Site	N	P	Quad	Date
1	* S. apiculata *	HZ	Rothwald, Kleiner Urwald *	1	L	82562	6 August 2012
2	* S. apiculata *	MK and HZ	Hintere Saigerin *	1	U	82533	18 July 2017
3	* S. apiculata *	MK and HZ	Toplitzsee *	1	St	83492	28 July 2017
4	* S. apiculata *	MK and HZ	Rothwald, Großer Urwald *	4	L	82562	10 July 2019
5	* S. apiculata *	MK and HZ	Lahnsattel	2	L	82591	20 June 2019
6	* S. apiculata *	MK and HZ	Brutgern *	1	S	83452	7 July 2020
7	* S. apiculata *	MK and HZ	Grießbachkessel *	1	S	82453	8 July 2020
8	* S. apiculata *	MK and HZ	Zellerbrunnbach *	1	St	82562	16 July 2020
9	* S. apiculata *	MK and HZ	Zellerbrunn-Dürradmer *	2	St	82562	17 July 2020
10	* S. apiculata *	MK and HZ	Josefsgraben *	3	St	83534	4 July 2022
1	* S. carinthiaca *	MK and HZ	Rothwald, Großer Urwald *	3	L	82562	9 July 2019
2	* S. carinthiaca *	MK and HZ	Groißnalm, Kalkalpen *	1	U	82523	18 July 2016
3	* S. carinthiaca *	MK and HZ	Kulisse, Kalkalpen *	1	U	82521	17 August 2016
4	* S. carinthiaca *	MK and HZ	Rettenbachtal *	1	St	83482	3 June 2017
5	* S. carinthiaca *	MK and HZ	Mitterwand *	1	St	83482	5 June 2017
6	* S. carinthiaca *	MK and HZ	Koppentraun left bank	1	St	84481	23 July 2019
7	* S. carinthiaca *	MK and HZ	Kammersee *	1	St	83494	25 July 2017
8	* S. carinthiaca *	MK and HZ	Toplitzsee *	1	St	83492	28 July 2017
9	* S. carinthiaca *	MK and HZ	Krumme Steyerling *	2	U	82521	7 June 2019
10	* S. carinthiaca *	MK and HZ	Lahnsattel	1	L	82591	21 June 2019
11	* S. carinthiaca *	MK and HZ	Grimmingbach N Gnanitzalm *	1	St	84501	24 July 2019
12	* S. carinthiaca *	MK and HZ	Haselschlucht, Kalkalpen *	1	U	82522	7 June 2020
13	* S. carinthiaca *	MK and HZ	Brutgern *	1	S	83452	7 July 2020
14	* S. carinthiaca *	MK and HZ	Grießbachkessel *	3	S	82453	8 July 2020
15	* S. carinthiaca *	MK and HZ	Ufer des Schallerbachs *	3	St	82564	13 July 2020
16	* S. carinthiaca *	MK and HZ	Salza, Prescenyriegel *	1	St	83564	13 July 2020
17	* S. carinthiaca *	MK and HZ	Zellerbrunnbach *	3	St	82562	16 July 2020
18	* S. carinthiaca *	MK and HZ	Josefsgraben, S Kropfbründl, Gesäuse *	1	St	83534	4 July 2022
19	* S. carinthiaca *	MK and HZ	Wasserfallweg, Gesäuse *	1	St	84532	5 July 2022
20	* S. carinthiaca *	MK and HZ	Ebner-Klamm: Schlucht 1, Gesäuse *	1	St	84534	7 July 2022
1	* S. scapanioides *	MK and HZ	Groißnalm, Kalkalpen *	1	U	82523	16 July 2016
2	* S. scapanioides *	MK and HZ	Krumme Steyerling *	1	U	82521	7 June 2019
3	* S. scapanioides *	MK and HZ	Geißlucke, Kalkalpen *	1	U	82522	10 June 2019
4	* S. scapanioides *	MK and HZ	Lahnsattel	1	L	82591	21 June 2019
5	* S. scapanioides *	MK and HZ	Wasserlochklammm, Wildalpen *	1	St	82553	13 July 2019
6	* S. scapanioides *	MK and HZ	Ufer des Schallerbachs, Wildalpen *	1	St	82564	13 July 2020
7	* S. scapanioides *	MK and HZ	Zellerbrunnbach, Wildalpen *	1	St	82562	16 July 2020
8	* S. scapanioides *	MK and HZ	Ebner-Klamm: Schlucht 1, Gesäuse *	2	St	84534	7 July 2022
9	* S. scapanioides *	MK and HZ	Ebner-Klamm: Schlucht 2, Gesäuse *	1	St	84534	7 July 2022
10	* S. scapanioides *	MK and HZ	Rohr-Bach, Gesäuse *	1	St	84532	8 July 2022

Abbreviations are as follows: Finder: MK = Michaela Kropik, HZ = Harald G. Zechmeister; Site: * = Protected area; N = Number of colonized logs; P = Austrian province, L = Lower Austria, S = Salzburg, St = Styria, U = Upper Austria; Quad = Quadrant of the Austrian Flora Mapping; Date = Date of discovery.

**Table A2:** Previously published reports of *S. apiculata*, *S. carinthiaca*, and *S. scapanioides* in Austria.

	Species	Finder	Site	P	Published in
1	* S. apiculata *	GS	Molln, Bodinggraben	U	[64]
2	* S. apiculata *	HK and AS	Stubalpe, Waldensteiner Graben	C	[6]
3	* S. apiculata *	HK and AS	Saualpe, Woisbachgraben	C	[6]
4	* S. apiculata *	K and K	Gailtaler Alpen, W Greifenburg	C	[6]
5	* S. apiculata *	HK	Karnische Alpen, Garnitzenklamm	C	[6]
6	* S. apiculata *	HK and AS	Karawanken, Strugarzagraben	C	[6]
7	* S. apiculata *	K and K	Zinkenbachgraben	S	[6]
8	* S. apiculata *	CS	Königsbachtal S Wolfgangsee	S	[6]
9	* S. apiculata *	B	Tannheim, Haldensee	T	[6]
10	* S. apiculata *	MR	Kleinwalsertal, Breitachtal	V	[6]
1	* S. carinthiaca *	GS	Haselschlucht	U	[64]
2	* S. carinthiaca *	HK	Ladritscher Tobel,	V	[65]
3	* S. carinthiaca *	HK	Vermielbach St. Gallenkirch	V	[65]
4	* S. carinthiaca *	HK	Hohe Tauern, Gößnitzfall	C	[6]
5	* S. carinthiaca *	HK and HvM	Kolmitzgraben bei Mörtschach	C	[6]
6	* S. carinthiaca *	HK	Lodronsteig im Maltatal	C	[6]
7	* S. carinthiaca *	HKandHvM	Lamnitzgraben	C	[6]
8	* S. carinthiaca *	HK	Fuggeralm, Flattnitz	C	[6]
9	* S. carinthiaca *	HK	Sommeraugraben, Reichenfels	C	[6]
10	* S. carinthiaca *	HK	Fraßgraben, Koralm	C	[6]
11	* S. carinthiaca *	HK	Kirchbachgraben N Kirchbach	C	[6]
12	* S. carinthiaca *	HK and HvM	Grießbachschlucht S Würmlach	C	[6]
13	* S. carinthiaca *	HK and MS	Kuplitzklamm SE Eisenkappl	C	[6]
14	* S. carinthiaca *	CS	Königsbachtal S Wolfgangsee	S	[6]
15	* S. carinthiaca *	K and K	Schwarzwand bei Hüttschlag	S	[6]
16	* S. carinthiaca *	HK	Gragger Schlucht SW Neumarkt	St	[6]
1	* S. scapanioides *	GS	Höllenleitenbachgraben/Großraming	U	[64]
2	* S. scapanioides *	HK and HvM	Marchgraben E Weißensee	C	[6]
3	* S. scapanioides *	HK and AS	Karawanken, Strugarzagraben	C	[6]
4	* S. scapanioides *	HK and AS	Eselgraben im Loibltal	C	[6]
5	* S. scapanioides *	HK and MS	Kuplitzklamm SE Eisenkappl	C	[6]

Abbreviations are as follows: Finder: AS = Adi Schriebl, B = Biedermann, CS = Christian Schröck, GS = Gerhard Schlüsslmayr, HK = Heribert Köckinger, HvM = Hubert van Melik, K and K = Koppe and Koppe, MR = Markus Reimann, MS = Michael Suanjak; P = Austrian province, C = Carinthia, S = Salzburg, St = Styria, U = Upper Austria, V = Vorarlberg.

## References

[1] Potemkin A.D. (2002). Phylogenetic system and classification of the family Scapaniaceae Mig. emend. Potemkin (Hepaticae). Ann. Bot. Fenn..

[2] Heinrichs J., Bombosch A., Feldberg K., Kreier H.P., Hentschel J., Eckstein J., Long D., Zhu R.L., Schafer-Verwimp A., Schmidt A.R. (2012). A phylogeny of the northern temperate leafy liverwort genus Scapania (Scapaniaceae, Jungermanniales). Mol. Phylogenetics Evol..

[3] Hodgetts N., Cálix M., Englefield E., Fettes N., Criado G.M., Patin L., Nieto A., Bergamini A., Bisang I., Baisheva E. (2019). A Miniature World in Decline: European Red List of Mosses, Liverworts and Hornworts.

[4] Müller K. (1957). Die Lebermoose Europas: Eine Gesamtdarstellung der Europäischen Arten.

[5] Schuster R.M. (1974). The Hepaticae and Anthocerotae of North America East of The Hundredth Meridian.

[6] Köckinger H. (2017). Die Horn- und Lebermoose Österreichs (Anthocerotophyta und Marchantiophyta). Catalogus Florae Austriae, II. Teil, Heft 2.

[7] Hodgetts N., Lockhart N. (2020). Checklist and Country Status of European Bryophytes—Update 2020.

[8] Kucera J., Vána J., Hradílek Z. (2012). Bryophyte flora of the Czech Republic: Updated checklist and Red List and a brief analysis. Preslia.

[9] Martincic A. (2016). Updated Red List of Bryophytes of Slovenia. Hacquetia.

[10] Mišíková K., Godovičová K., Širka P., Šoltés R. (2021). Checklist and red list of hornworts (Anthocerotophyta) and liverworts (Marchantiophyta) of Slovakia. Biologia.

[11] Caspari S., Dürhammer O., Sauer M., Schmidt C., Metzing D., Hofbauer N., Ludwig G., Matzke-Hajek G. (2013). Rote Liste und Gesamtartenliste der Moose (Anthocerotophyta, Marchantiophyta und Bryophyta) Deutschlands. Rote Liste Gefahrdeter Tiere, Pflanzen und Pilze Deutschlands. Band 7: Pflanzen.

[12] Senn H., Lehrmittelverlag A. (2000). Die Moose des Furstentums Liechtenstein. Naturkundliche Forschung im Furstentum Liechtenstein.

[13] Konstantinova N. (2019). Scapania Apiculata (Europe Assessment). https://www.iucnredlist.org/species/87522879/87758862.

[14] Schröck C., Bisang I., Caspari S., Hedenäs L., Hodgetts N., Kiebacher T., Kučera J., Ştefănuţ S., Vana J. (2019). Scapania Carinthiaca (Europe Assessment). https://www.iucnredlist.org/species/87523480/87741209.

[15] Schröck C., Bisang I., Caspari S., Hedenäs L., Hodgetts N., Kiebacher T., Kučera J., Ştefănuţ S., Vana J. (2019). Scapania Scapanioides (Europe Assessment). https://www.iucnredlist.org/species/88289151/88382772.

[16] Hassel K., Jordal J.B., Gaarder G. (2006). Scapania apiculata, S. carinthiaca and S. glaucocephala, three rare liverworts (Marchantiophyta) on decaying logs in stream crevices and small watercourses. Blyttia.

[17] Kropik M., Zechmeister H.G., Moser D., Bernhardt K.G., Dullinger S. (2021). Deadwood volumes matter in epixylic bryophyte conservation, but precipitation limits the establishment of substrate-specific communities. For. Ecol. Manag..

[18] Qiao Y., Zheng H., Li L., Zhang J., Li Y., Li S., Zhu R., Zhou J., Zhao S., Jiang Y. (2018). Terpenoids with vasorelaxant effects from the Chinese liverwort Scapania carinthiaca. Bioorganic Med. Chem..

[19] Peters K., Treutler H., Döll S., Kindt A.S.D., Hankemeier T., Neumann S. (2019). Chemical diversity and classification of secondary metabolites in nine bryophyte species. Metabolites.

[20] Proctor M.C.F., Smith A.J.E. (1982). Physiological Ecology: Water Relations, Light and Temperature Responses, Carbon Balance. Bryophyte Ecology.

[21] Proctor M.C.F., Oliver M.J., Wood A.J., Alpert P., Stark L.R., Cleavitt N.L., Mishler B.D. (2007). Desiccation-tolerance in bryophytes: A review. Bryologist.

[22] Kropik M., Zechmeister H.G., Moser D. (2021). Climate Variables Outstrip Deadwood Amount: Desiccation as the Main Trigger for Buxbaumia viridis Occurrence. Plants.

[23] Dilks T.J.K., Proctor M.C.F. (1974). Pattern of recovery of bryophytes after desiccation. J. Bryol..

[24] Köckinger H., Schröck C. (2017). Rote Liste der Moose Kärntens.

[25] Zechmeister H.G., Hagel H., Gendo A., Osvaldik V., Patek M., Prinz M., Schröck C., Köckinger H. (2013). Die Rote Liste der Moose Niederösterreichs. Wiss. Mitteilungen Des Niederösterreichischen Landesmus..

[26] Schröck C., Köckinger H., Schlüsselmayr G. (2014). Katalog und Rote Liste der Moose Oberösterreichs. Stapfia.

[27] Schröck C., Köckinger H., Amann G., Zechmeister H.G. (2013). Rote Liste gefährdeter Moose Vorarlbergs.

[28] Taborska M., Privetivy T., Vrska T., Odor P. (2015). Bryophytes associated with two tree species and different stages of decay in a natural fir-beech mixed forest in the Czech Republic. Preslia.

[29] Odor P., Van Hees A.F.M. (2004). Preferences of Dead Wood Inhabiting Bryophytes for Decay Stage, Log Size and Habitat Types in Hungarian Beech Forests. J. Bryol..

[30] Hassel K. (2018). What is the effect of small-scale hydroelectric power plants on bryophytes growing on lying decaying wood? A population study of Scapania apiculata. Blyttia.

[31] Hofmeister J., Hosek J., Brabec M., Dvorak D., Beran M., Deckerova H., Burel J., Kriz M., Borovicka J., Bet’ak J. (2015). Value of old forest attributes related to cryptogam species richness in temperate forests: A quantitative assessment. Ecol. Indic..

[32] Sabatini F.M., Burrascano S., Keeton W.S., Levers C., Lindner M., Potzschner F., Verkerk P.J., Bauhus J., Buchwald E., Chaskovsky O. (2018). Where are Europe’s last primary forests?. Divers. Distrib..

[33] Lassauce A., Paillet Y., Jactel H., Bouget C. (2011). Deadwood as a surrogate for forest biodiversity: Meta-analysis of correlations between deadwood volume and species richness of saproxylic organisms. Ecol. Indic..

[34] Paillet Y., Berges L., Hjalten J., Odor P., Avon C., Bernhardt-Romermann M., Bijlsma R.J., De Bruyn L., Fuhr M., Grandin U. (2010). Biodiversity Differences between Managed and Unmanaged Forests: Meta-Analysis of Species Richness in Europe. Conserv. Biol..

[35] Müller J., Bütler R. (2010). A review of habitat thresholds for dead wood: A baseline for management recommendations in European forests. Eur. J. For. Res..

[36] Moning C., Held M., Moshammer R., Muller J. (2010). Okologische Schwellenwerte in Bergmischwaldern als Basis fur forstliche Naturschutzkonzepte. Naturschutz Und Landschaftsplanung.

[37] Longton R.E., Schuster R.M., Schuster R.M. (1983). Reproductive biology. New Manual of Bryology.

[38] Kimmerer R.W. (1991). Reproductive ecology of Tetraphis pellucida 2—Differential success of sexual and asexual propagules. Bryologist.

[39] Niklas K.J., Cobb E.D. (2017). The evolutionary ecology (evo-eco) of plant asexual reproduction. Evol. Ecol..

[40] Hanski I. (1991). Single-species metapopulation dynamics—Concepts, models and observations. Biol. J. Linn. Soc..

[41] Söderström L., Herben T., Longton R.E. (1997). Dynamics of Bryophyte Metapopulations. Population Studies.

[42] Laaka-Lindberg S., Korpelainen H., Pohjamo M. (2003). Dispersal of asexual propagules in bryophytes. J. Hattori Bot. Lab..

[43] Löbel S., Rydin H. (2009). Dispersal and life history strategies in epiphyte metacommunities: Alternative solutions to survival in patchy, dynamic landscapes. Oecologia.

[44] Chmielewski M.W., Eppley S.M. (2019). Forest Passerines as a Novel Dispersal Vector of Viable Bryophyte Propagules. Proc. R. Soc. B Biol. Sci..

[45] Kropik M., Zechmeister H.G., Fuxjager C. (2020). The Fate of Bryophyte Sporophytes-Phenology and Vectors of Buxbaumia viridis in the Kalkalpen National Park, Austria. Plants.

[46] Zechmeister H.G., Köckinger H., Kropik M. (2019). Erfassung ausgewählter Moose im Anhang II der FFH-Richtlinie in der Steiermark. Endbericht. https://www.verwaltung.steiermark.at/cms/dokumente/12104065_110669261/7be7d067/FFH_Moose_Steiermark_2021.pdf.

[47] Zechmeister H.G., Kropik M. (2022). Erfassung der FFH-Moose Mannia Triandra und Scapania Carinthiaca im Nationalpark Gesause. Endbericht. https://www.parcs.at/npg/pdf_public/2022/51257_20220919_115159_Endbericht_FFH-Moose_Zechmeister_2022_inkl.Metadaten_frParcs.pdf.

[48] Zechmeister H.G., Kropik M., Schröck C. (2017). Erfassung der Moose im Anhang II Der FFH-Richtlinie im Nationalpark Kalkalpen. https://www.kalkalpen.at/de/Erfassung_der_FFH_Moose_im_Nationalpark_Kalkalpen.

[49] Kropik M. (2021). Bryophytes on Deadwood—Drivers of Diversity and Distribution. Ph.D. Thesis.

[50] Köckinger H., Schröck C., Krisai R., Zechmeister H.G. (2020). Checkliste der Moose Österreichs. http://cvl.univie.ac.at/projekte/moose.

[51] Damsholdt K. (2002). Illustrated Flora of Nordic Liverworts and Hornworts.

[52] Paton J.A. (1999). The Liverwort Flora of the British Isles.

[53] Buch H. (1928). Die Scapanien Nordeuropas und Sibiriens. II. Systematischer Teil. Comment. Biol. Soc. Sci. Fenn..

[54] Crandall-Stotler B., Stotler R.E., Long D.G. (2009). Phylogeny and classification of the Marchantiophyta. Edinb. J. Bot..

[55] Potemkin A.D. (1999). An analysis of the practical taxonomy of some critical northern species of Scapania (Scapaniaceae, Hepaticae). Bryologist.

[56] Hiebl J., Frei C. (2016). Daily temperature grids for Austria since 1961—Concept, creation and applicability. Theor. Appl. Climatol..

[57] Hiebl J., Frei C. (2018). Daily precipitation grids for Austria since 1961—Development and evaluation of a spatial dataset for hydroclimatic monitoring and modelling. Theor. Appl. Climatol..

[58] Proctor M.C.F., Tuba Z. (2002). Poikilohydry and homoihydry: Antithesis or spectrum of possibilities?. New Phytol..

[59] He X.L., He K.S., Hyvonen J. (2016). Will bryophytes survive in a warming world?. Perspect. Plant Ecol. Evol. Syst..

[60] Wood A.J. (2007). Invited essay: New frontiers in bryology and lichenology—The nature and distribution of vegetative desiccation-tolerance in hornworts, liverworts and mosses. Bryologist.

[61] Düggelin C., Kellner M. (2017). Schweizerisches Forstinventar. Feldaufnahme-Anleitung 2017. https://www.dora.lib4ri.ch/wsl/islandora/object/wsl:16234.

[62] Lachat T., Brang P., Bolliger M., Bollmann K., Brändli U., Bütler R., Herrmann S., Schneider O., Wermelinger B. (2019). Totholz im Wald. Entstehung, Bedeutung und Förderung. Merkbl. FÜR Die Prax..

[63] (2023). R: A Language and Environment for Statistical Computing.

[64] Schlüsselmayr G. (2005). Soziologische Moosflora des südöstlichen Oberösterreich. Stapfia.

[65] Amann G., Köckinger H., Reimann M., Schröck C., Zechmeister H.G. (2013). Bryofloristische Ergebnisse der Mooskartierung in Vorarlberg. Stapfia.

